# Emerging Approaches in Glioblastoma Treatment: Modulating the Extracellular Matrix Through Nanotechnology

**DOI:** 10.3390/pharmaceutics17020142

**Published:** 2025-01-21

**Authors:** Miguel Horta, Paula Soares, Catarina Leite Pereira, Raquel T. Lima

**Affiliations:** 1 i3S—Instituto de Investigação e Inovação em Saúde, University of Porto, Rua Alfredo Allen 208, 4200-135 Porto, Portugal; mhorta@i3s.up.pt (M.H.); psoares@ipatimup.pt (P.S.); 2 IPATIMUP—Instituto de Patologia e Imunologia Molecular, University of Porto, Rua Júlio Amaral de Carvalho 45, 4200-135 Porto, Portugal; 3 FMUP—Faculty of Medicine, University of Porto, Alameda Prof. Hernâni Monteiro, 4200-319 Porto, Portugal; 4 INEB—Instituto Nacional de Engenharia Biomédica, University of Porto, Rua Alfredo Allen 208, 4200-135 Porto, Portugal

**Keywords:** nanotechnology, nanoparticles, glioblastoma (GB), tumor microenvironment (TME), extracellular matrix (ECM)

## Abstract

Glioblastoma’s (GB) complex tumor microenvironment (TME) promotes its progression and resistance to therapy. A critical component of TME is the extracellular matrix (ECM), which plays a pivotal role in promoting the tumor’s invasive behavior and aggressiveness. Nanotechnology holds significant promise for GB treatment, with the potential to address challenges posed by both the blood-brain barrier and the GB ECM. By enabling targeted delivery of therapeutic and diagnostic agents, nanotechnology offers the prospect of improving treatment efficacy and diagnostic accuracy at the tumor site. This review provides a comprehensive exploration of GB, including its epidemiology, classification, and current treatment strategies, alongside the intricacies of its TME. It highlights nanotechnology-based strategies, focusing on nanoparticle formulations such as liposomes, polymeric nanoparticles, and gold nanoparticles, which have shown promise in GB therapy. Furthermore, it explores how different emerging nanotechnology strategies modulate the ECM to overcome the challenges posed by its high density, which restricts drug distribution within GB tumors. By emphasizing the intersection of nanotechnology and GB ECM, this review underscores an innovative approach to advancing GB treatment. It addresses the limitations of current therapies, identifies new research avenues, and emphasizes the potential of nanotechnology to improve patient outcomes.

## 1. Glioblastoma: Epidemiology and Classification

Glioblastoma (GB) is the most aggressive and highly malignant brain tumor in adults, presenting a poor prognosis. Its incidence varies depending on the population studied, mostly ranging from 3 to 4.5 per 100,000 people annually [1,2,3], accounting for approximately 50% of all malignant central nervous system (CNS) tumors [4]. GB shows a slight male predominance, with a male to female ratio of about 1.6:1 [5]. Although the incidence increases with age, it can occur across all adult age groups [1]. While CNS tumors are common in children, according to the most recent World Health Organization (WHO) guidelines, GB is no longer a possible diagnosis in this age group; instead, it is considered a diffuse pediatric glioma [6].

**Figure 1:**
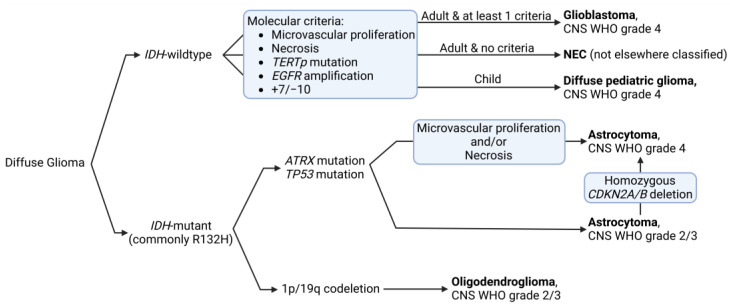
Glioma classification according to the WHO 2021 classification. CNS WHO grades: (1) Generally indolent tumors with favorable prognosis, (2) Low-grade tumors with potential for recurrence, (3) Malignant tumors with increased mitotic activity, (4) Highly malignant tumors with rapid progression.

As our knowledge of GB improves, so do our methods for diagnosing, classifying, and treating the disease [7]. Recent changes in tumor classification, driven by new insights from molecular profiling, have significantly influenced how to diagnose and treat GB, highlighting the clinical importance of these advancements (Figure 1) [8]. Under the most recent 2021 WHO classification of tumors of the CNS, which has significantly redefined GB, GB is strictly defined as an isocitrate dehydrogenase (*IDH*)-wildtype, WHO grade 4, tumor. This reclassification emphasizes the distinct biological behavior and more favorable prognosis of *IDH*-mutant tumors compared to their *IDH*-wildtype counterparts [1,6,9,10]. Furthermore, for the diagnosis of GB (*IDH*-wildtype WHO grade 4) at least one of the following criteria is now required: (i) microvascular proliferation or necrosis on histological examination; (ii) telomerase reverse transcriptase (*TERT*) gene promoter mutations; (iii) epithelial growth factor (EGF) receptor (*EGFR*) gene amplification; (iv) combined gain of chromosome 7 and loss of chromosome 10 (+7/−10) [11]. This integrated approach, combining histological and molecular features, provides a more accurate and clinically relevant diagnosis. Notably, this new classification no longer allows for the use of “not otherwise specified” (NOS) in GB diagnosis, further underscoring the importance of molecular testing in modern neuro-oncology [12]. Moreover, the “not elsewhere classified” (NEC) designation is now used when a tumor does not meet the specific GB criteria but still appears to be a diffuse astrocytic glioma without *IDH* mutation [13].

The prognosis for GB remains poor, with median survival varying based on molecular subtypes, which are defined by different transcription profiles [14]. The mesenchymal subtype is characterized by extensive necrosis and inflammation, exhibiting transcription profiles similar to mesenchymal tissues. The proneural subtype, considered milder, is characterized by the expression of genes involved in neurogenesis and oligodendrocyte development. The classical subtype, in between, shows features similar to astrocytes, hence the “classical” name reflecting typical GB characteristics [15]. The mesenchymal subtype has the worst prognosis, with a median survival of about 11.5 months, followed by the classical subtype at 14.7 months, and the proneural subtype at 17.0 months [16]. These survival differences highlight the clinical relevance of molecular subtyping in GB, which will be discussed in more detail later. Although there are several risk factors for GB, exposure to high-dose ionizing radiation and certain rare genetic conditions (e.g., Lynch syndrome, Neurofibromatosis type 1, Li-Fraumeni syndrome) have been associated with increased incidence [17,18]. However, the majority of GB cases occur sporadically without any known risk factors [19]. Anatomically, GBs are typically supratentorial, with rare cases in the cerebellum or spine [20,21]. They most commonly affect the frontal and temporal lobes, followed by the parietal and occipital lobes, with a slight preference for the right hemisphere [22]. Tumor location can significantly impact surgical resection and, consequently, patient outcomes [23,24].

## 2. Current Treatment Options

### 2.1. First-Line—Stupp Protocol

The “Stupp protocol”, introduced in 2005, is the standard of care for treating newly diagnosed GB cases [25]. Named after the Swiss oncologist Roger Stupp, this treatment regimen improved survival outcomes for GB patients compared to previous approaches. The protocol consists of maximal safe surgical resection followed by two main phases, concurrent chemoradiotherapy followed by adjuvant chemotherapy. During the first phase, patients receive radiotherapy (RT) with a total dose of 60 Gy, delivered in 30 fractions of 2 Gy each over 6 weeks (5 days per week). Simultaneously, patients take oral temozolomide (TMZ), an alkylating chemotherapy agent, at a dose of 75 mg/m^2^ of body surface area, daily, seven days a week throughout the RT period. During the second phase, after a 4-week break once completing RT, patients undergo six cycles of TMZ monotherapy. Each cycle lasts 28 days, with TMZ taken for the first 5 days at a dose of 150–200 mg/m^2^ [26].

The Stupp protocol demonstrated an improvement in survival compared to RT alone. In the original study, the median overall survival increased from 12.1 months with RT alone to 14.6 months with the combined approach. More importantly, the 2-year survival rate improved from 10.4% to 26.5% [27]. Despite these improvements, the prognosis for GB remains poor. Nevertheless, this protocol is most effective in patients under 70 years of age, with good performance status, and whose tumors exhibit methylguanine methyltransferase (*MGMT*) promoter methylation, a biomarker associated with better TMZ treatment response [28]. Moreover, side effects of the Stupp protocol can be significant, with the most common being fatigue, nausea, and myelosuppression, and the most severe complications including neutropenia, thrombocytopenia, lymphopenia, and increased risk of opportunistic infections. To reduce infection risks, patients with these complications often receive antibiotic prophylaxis during treatment [29]. While the Stupp protocol remains the backbone standard of care for GB, ongoing research seeks to further improve patient outcomes.

### 2.2. Second-Line Treatments

Following Stupp protocol failure, with frequent tumor relapse, there is no universally accepted standard for second-line treatment. The management of recurrent GB remains a therapeutic challenge, with several strategies being explored. Tumor reoperation is considered for patients with large, symptomatic tumors, but its benefits remain controversial and depend on patient selection [30,31,32]. Another strategy, reirradiation, presents options including stereotactic radiosurgery, hypofractionated stereotactic RT, conventionally fractioned external RT, or brachytherapy. Decision depends on factors such as time since initial radiation and tumor characteristics [33,34,35]. A third option is a TMZ rechallenge, which is considered for initial responders with prolonged treatment-free intervals. Various dosing schedules have been explored, but overall survival rarely exceeds 12 months [36,37,38,39,40]. Treatment with nitrosoureas, such as carmustine, lomustine, nimustine, and fotemustine, have also shown limited efficacy in recurrent GB, with median overall survival rarely exceeding 12 months [41,42,43,44]. Bevacizumab, a Food and Drug Administration (FDA)-approved anti-angiogenic antibody, has often been employed as a strategy combined with other agents, such as irinotecan or lomustine. While it has been shown to improve progression-free survival, impact on overall survival is still debated [45,46,47,48]. Nevertheless, recent research has indicated that high vascular endothelial growth factor (VEGF)-A expression is associated with improved progression-free survival in recurrent GB patients treated with bevacizumab, suggesting potential for a therapeutic improvement through biomarker-guided treatment selection [49]. The multi-kinase inhibitor Regorafenib has shown potential benefits in the REGOMA trial, but more comprehensive data are still needed due to its relatively recent introduction in clinical practice in 2020 [50,51,52]. Other tyrosine kinase inhibitors, such as cabozantinib, dasatinib, and entrectinib, are currently being evaluated, particularly in molecularly selected populations. However, their efficacy as monotherapies has been limited, mainly due to lack of tumor specificity and poor blood-brain barrier (BBB) permeability [53,54,55]. Over the last decade, immunotherapy, particularly checkpoint inhibitors, has emerged as a potential therapy for GB. While its efficacy has been limited in unselected GB populations, research continues to address its potential in patient specific subgroups [56,57,58,59]. Also, Tumor Treating Fields (TTFields) have emerged as a promising second-line treatment option in clinical practice. This device-based therapy delivers alternating electric fields to tumor sites, promoting cancer cell death by disrupting microtubule alignment during cell division [60,61,62]. TTFields have demonstrated safety and efficacy in clinical trials, leading to FDA approval for GB and malignant pleural mesothelioma. As TTFields continue to show promise, ongoing clinical trials are exploring its use in lung, ovarian, pancreatic, and other cancers, signaling a growing integration of this innovative approach into standard cancer care protocols [63].

GB treatment goals in the recurrent setting are often palliative, aiming to improve or maintain quality of life while potentially extending survival. The choice of second-line treatments for recurrent GB is highly individualized and depends on various factors, including the patient’s age, performance status, tumor molecular profile, response to initial therapy, and time to recurrence [64]. Despite the availability of the referred options, the prognosis for recurrent GB remains poor, with median overall survival typically ranging from 6 to 12 months in a clinical setting, with a very modest increase to approximately 15 months for clinical trial participants [65,66,67,68]. This underscores the urgent need for more effective therapies and highlights the importance of ongoing research in this challenging cancer type.

## 3. Glioblastoma and Its Microenvironment—Cellular and Molecular Features

**Figure 2:**
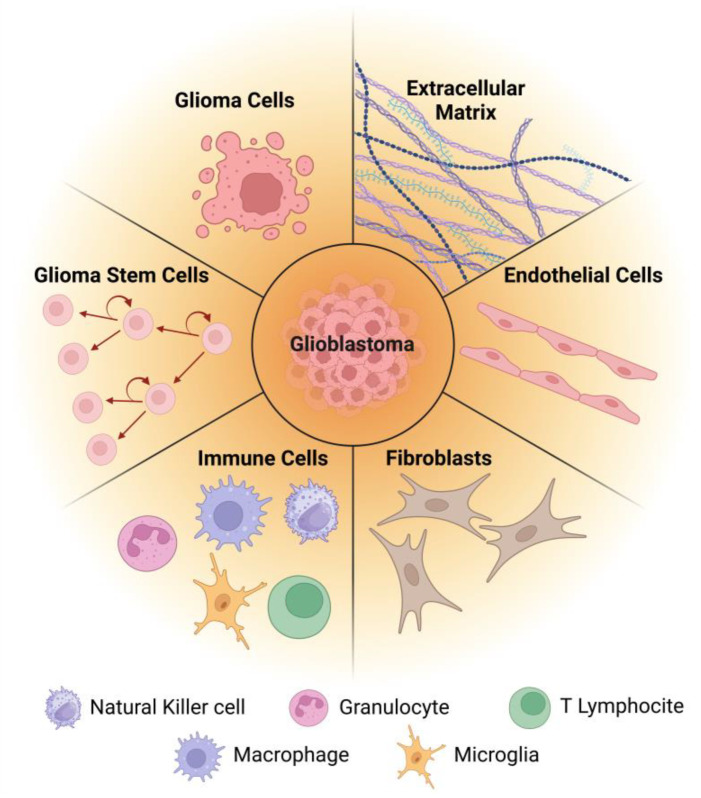
Cellular and non-cellular components of GB and its microenvironment.

Adding to its own cellular and molecular features, GB is characterized by a complex and dynamic tumor microenvironment (TME) that plays a crucial role in tumor progression, invasion, and therapy resistance [69]. The TME is composed of various cellular and molecular components (Figure 2) that intricately interact to support tumor growth and promote immune evasion [70]. These interactions are key to understanding the mechanisms of GB progression and therapy resistance. As such, gaining a deeper understanding of the TME is essential for developing effective therapeutic strategies.

### 3.1. Glioma Cells

The primary cellular component of GB tumors consists of malignant glial cells, which exhibit significant inter- and intra-tumoral heterogeneity [71]. This heterogeneity was classified into the previously referred three molecular subtypes, classical, mesenchymal, and proneural, based on transcriptomic analyses from The Cancer Genome Atlas (TCGA) [72]. A fourth one was initially proposed, the neural subtype, but later excluded as it was based on a misidentification caused by cellular cross contamination [16].

The classical subtype is the least common among GB tumors, accounting for approximately 20% of all GBs [73]. It is characterized by *EGFR* amplification in nearly all tumors (97%), and some harboring *EGFR* mutations [74,75]. Consistent genetic alterations included chromosome 7 amplification paired with chromosome 10 loss and focal 9p21.3 homozygous deletion targeting cyclin-dependent kinase inhibitor 2A (*CDKN2A*), which mostly co-occur with *EGFR* amplification [15]. Notably, tumor protein p53 (*TP53*) mutations are rare in the classical subtype despite being the most frequently mutated gene in GB overall [76]. Furthermore, the transcriptomic analyses showed an overexpression of Sonic-hedgehog (smoothened (SMO), growth arrest-specific protein 1 (GAS1), and GLI family zinc finger 2 (GLI2)) and Notch pathway (neurogenic locus notch homolog protein 3 (NOTCH3), jagged 1 (JAG1), and beta-1,3-*N*-acetylglucosaminyltransferase lunatic fringe (LFNG)) agents, along with high expression of the neural precursor marker nestin [77,78]. Patients with the classical subtype benefit from aggressive RT and chemotherapy [78].

The prevalence of the mesenchymal subtype ranges from 30% to 50% of all GB tumors depending on the population studied and the molecular classification methods used [73,79]. It frequently exhibits neurofibromin (*NF1*) mutations and deletions at 17q11.2 (further affecting NF1 expression), often co-occurring with phosphatase and tensin homolog (*PTEN*) mutations and phosphatidylinositol 3-kinase (PI3K)/AKT pathway enrichment [15,80]. Mesenchymal markers such as chitinase-3-like protein 1 (CHI3L1) and proto-oncogene tyrosine kinase MET are highly expressed, along with astrocytic markers like cluster of differentiation (CD) 44 and proto-oncogene tyrosine kinase MER, indicating potential epithelial-to-mesenchymal transition [15]. Gene expression analysis revealed activation of tumor necrosis factor and nuclear factor kappa-light-chain-enhancer of activated B cells (NF-κB) pathways, correlating with extensive necrosis and inflammatory infiltrates characteristic of this subtype [74,78]. Clinically, while responsive to aggressive RT and chemotherapy, patients with mesenchymal GB have the worst prognosis among all GB subtypes [14].

The proneural subtype, slightly less common than the mesenchymal subtype, accounts for approximately a third of GB tumors [73]. It is characterized by distinct genetic alterations, primarily involving the platelet-derived growth factor receptor A (*PDGFRA*) gene [78]. This subtype was associated with a gene expression profile linked to oligodendrocyte development and neurogenesis, reflecting its unique biological characteristics. The proneural subtype was also characterized by frequent *TP53* mutations and loss of heterozygosity [15]. Clinically, patients with this subtype generally have a better prognosis compared to those with other subtypes, particularly in younger populations [14,76,77,80].

A more recent perspective on GB heterogeneity suggests that genetic factors alone do not fully account for the diverse glioma cell composition observed within these tumors. An international team of researchers proposed that somatic mutations play a crucial role in shaping the tumor’s cellular landscape by selectively promoting certain cellular states over others [81]. This process results in a dynamic equilibrium of different cell populations within the tumor, contributing to its heterogeneous nature. Neftel et al. conducted a computational analysis that identified gene signatures converging into four recurring cellular states across multiple GB tumors [81]. These states were present in varying combinations of two to four in each patient, again denoting the heterogeneity of GB, being classified as astrocyte-like (AC-like), mesenchymal-like (MES-like), oligodendrocyte progenitor-like (OPC-like), and neural progenitor-like (NPC-like). OPC-like states were enriched in tumors with *PDGFRA* alterations, NPC-like states in those with cyclin-dependent kinase 4 (*CDK4*) alterations, and AC-like states in tumors with *EGFR* alterations. MES-like states were characterized by *NF1* alterations, extensive immune cell infiltration, and hypoxic conditions [81]. These cellular states aligned with the TCGA subtypes and corroborated findings from other studies suggesting that genomic aberrations alone do not fully explain GB heterogeneity [77,82]. Regardless, the heterogeneity of GB cell subtypes has significant clinical implications, with recent studies demonstrating that specific cellular compositions of tumors directly impact patient survival outcomes [83].

### 3.2. Glioma Stem Cells

A particularly concerning aspect of GB tumors is the presence of glioma stem cells (GSCs) in its TME, a subpopulation with self-renewal capacity that is thought to drive tumor initiation, recurrence, and therapy resistance [84]. Importantly, GSCs are now understood to represent a functional state rather than a distinct cell lineage [85]. This concept has been further supported by evidence showing that GB cells can dynamically acquire or lose stem-like characteristics in response to TME signals [86]. GSCs share features with normal neural stem cells, including stem cell marker expression such as nestin, sex determining region Y box 2 (SOX2), and CD133, as well as multi-lineage differentiation potential [85]. However, opposite to their normal counterpart, GSCs exhibit aberrant signaling pathway activation, enhancing survival, proliferation, and apoptosis resistance. These alterations confer GSCs a distinct advantage, enabling them to survive conventional therapies and contribute to tumor relapse, making them critical targets for developing more effective GB treatments [87]. In fact, evidence suggests that GSCs are more resistant to chemotherapy and RT than the bulk of tumor cells [85]. They possess enhanced deoxyribonucleic acid (DNA) repair mechanisms, activated survival pathways, and overexpress drug efflux transporters such as ATP-binding cassette (ABC) proteins [88]. Furthermore, GSCs often reside in specialized niches within the TME, which are characterized by hypoxia and supportive stromal cells. The niches provide protective signals that promote GSCs survival and maintenance, further shielding them from therapeutic interventions [89]. GSCs metabolism is regulated by several key signaling pathways, including Notch, Hedgehog, and Wnt. These pathways, essential for normal stem cell maintenance, are often dysregulated in cancer. Furthermore, transcription factors, such as SOX2, octamer-binding transcription factor 4 (OCT4), and c-Myc, are essential for maintaining GSCs pluripotency and proliferative capacity [90]. While GSCs are certainly influenced by TME cues, studies have also emphasized the reciprocal relationship between GSCs and their microenvironment, supporting and promoting glioma progression [91]. On one hand, TME-derived cytokines such as interleukin (IL)-6 and transforming growth factor beta (TGF-β) enhance GSCs self-renewal and survival [92]. On the other hand, GSCs have been shown to secrete factors that modulate the surrounding immune landscape, creating an immunosuppressive environment conducive to tumor growth. For example, GSCs can induce tumor-associated macrophages (TAMs) to adopt an M2-like phenotype, which suppresses immune responses and promotes tumor development [93]. This intricate interaction between GSCs and their microenvironment underscores the complexity of GB biology and highlights potential targets for therapeutic intervention.

### 3.3. Immune Cells

The immune component of GB represents approximately 50% of the tumor’s cellularity [94]. As previously mentioned, TAMs, which are differentiated from the resident microglia and peripheral monocytes, account for 30% of the tumor mass. These TAMs tend to adopt an M2 anti-inflammatory phenotype and have been shown to suppress CD8^+^ T cell activity, thereby promoting immune evasion [95]. TAMs are recruited by glioma-cell-derived factors, such as colony stimulating factor 1 (CSF-1), glial cell line-derived neurotrophic factor (GDNF), chemokine (CC motif) ligand 2 (CCL2), stromal cell-derived factor 1 (SFD-1), chemokine (CXC motif) ligand 1 (CXCL1), EGF, and granulocyte-macrophage colony-stimulating factor (GM-CSF) [96]. Once in the TME, TAMs secrete a range of molecules, including anti-inflammatory cytokines (IL4, IL10, TGFβ), angiogenesis factors (VEGF, IL8), and pro-tumorigenic growth factors (insulin-like growth factor 1 (IGF-1), EGF, and platelet-derived growth factor (PDGF)). These molecules significantly influence the GB TME and contribute to tumor progression and therapy resistance, ultimately leading to poorer prognosis for GB patients [94].

Another cell type within the GB TME is myeloid-derived suppressor cells (MDSCs), a diverse population of myeloid progenitor and precursor cells, including macrophages, granulocytes, and dendritic cells at various stages of differentiation [97]. Similar to TAMs, MDSCs impair the function of various T cell subsets, including natural killer T (NK) cells and cytotoxic T lymphocytes [98]. MDSCs also deplete essential amino acids from the TME, such as L-Arginine, and enhance the production of reactive oxygen species (ROS) to suppress T-cell activation and function, thereby contributing to the immune evasion of tumors. These changes in the TME create an unfavorable environment for T cells, leading to reduced immune responses against the tumor [99,100].

Lastly, NK cells, which are important to innate antitumor immunity through antigen-independent immune surveillance, were found to be the least abundant immune cell type in GB. Furthermore, their immune cytolytic activity is suppressed by major histocompatibility complex class I molecules expressed on GB cells [101].

### 3.4. Fibroblasts

Recent research has shed new light on the presence and significance of cancer-associated fibroblasts (CAFs) in GB, revealing their role in shaping the TME [102]. Being the primary extracellular matrix (ECM) producers, CAFs contribute significantly to the remodeling of the ECM in GB, promoting tumor growth and invasion. Their abundance correlates with higher tumor grades and activation of ECM remodeling pathways [102]. CAFs also secrete pro-tumorigenic factors, including fibronectin, which plays a critical role in promoting GB cell migration and invasion, thereby enhancing the tumor’s aggressive nature [102]. Furthermore, CAFs have been shown to promote resistance to TMZ through the secretion of CCL2, which activates the extracellular signal-regulated kinases (ERK) 1/2 signaling pathway in GB cells [103]. Interestingly, CAFs exhibit subtype-specific effects in GB [104]. They are more abundant in the mesenchymal subtype compared to other subtypes, while proneural GB cells appear to be more responsive to CAF signaling in terms of migratory and invasive behaviors. This heterogeneity in CAF distribution and influence across GB subtypes adds another layer of complexity to the TME [102]. CAFs have been shown to suppress anti-cancer immune responses in various cancers, highlighting their role in contributing to the immunosuppressive environment characteristic of GB [105].

### 3.5. Endothelial Cells

While not a part of the GB per se, endothelial cells, which encompass the perivascular niche, have been shown to be key components in the tumor’s aggressiveness, since angiogenesis is one of the hallmarks of GB recurrence, proliferation, and invasion [106]. In the GB TME, newly formed blood vessels are structurally fragile and prone to rupture, leading to a compromised BBB. This disruption causes an increased vascular permeability and edema, altering immune responses and the boundary between tumor and normal brain tissue, thus facilitating tumor invasiveness and presents challenges for effective treatment delivery [107].

The recruitment of endothelial cells to the GB site occurs through several mechanisms. Neoangiogenesis is the primary process, where GB cells secrete high levels of pro-angiogenic factors, particularly VEGF, stimulating the formation of new blood vessels from the sprouting of pre-existing ones. This process involves endothelial cell proliferation and migration towards the tumor [108]. Additionally, GBs recruit bone marrow-derived endothelial progenitor cells through vasculogenesis, which then differentiate into endothelial cells within the TME [109]. Lastly, GSCs also have the potential to transdifferentiate into endothelial cells, or to simply assemble into tubular-like structures mimicking blood vessels, further contributing to tumor vasculature [110].

Endothelial cells’ contribution to tumor progression is multifaceted. By forming new blood vessels, they ensure a steady supply of nutrients and oxygen to the rapidly growing tumor while enabling invasion through the vasculature [109]. Endothelial cells also create a specialized niche that promotes the self-renewal of GSCs by expressing NOTCH ligands that activate Notch signaling in adjacent GSCs, supporting their maintenance and proliferation [111]. Furthermore, endothelial cells facilitate bidirectional communication within the TME, exchanging extracellular vesicles with tumor cells, which can, in turn, promote angiogenesis, suppress the immune system, and confer drug resistance [108].

### 3.6. Extracellular Matrix

The non-cellular component of the GB TME is the ECM. The brain has an unique ECM composition, which accounts for approximately 20% of its volume [112]. It is comprised of various components, including, but not limited to: collagen IV; glycoproteins, such as fibronectin, laminins, and tenascins; glycosaminoglycans, such as hyaluronic acid (HA); and proteoglycans, such as the lectican family, phosphacan, and neuron-glial antigen 2 [113]. While sharing many components with the normal brain, the ECM of GB exhibits distinct characteristics that influence tumor behavior. Besides the fact that GB cells overexpress various ECM components, including HA, brevican, tenascin-C (TN-C), and fibronectin, they also show increased expression of specific integrins and receptors, which promote cell adhesion, migration, and invasion [114].

Notably, HA levels correlate with tumor malignancy, both increasing in parallel. HA, along with fibronectin, has been shown to promote the invasiveness of GB cells [115,116]. GB ECM is more condensed and less flexible compared to normal brain tissue due to overexpression of these ECM components, potentially hindering the diffusion of therapeutic agents and neuroactive molecules [117]. The tumor also induces altered protein synthesis in surrounding normal tissues, promoting ECM degradation at the invasive front while increasing ECM component production in nearby areas [118,119]. GB cells can actively migrate along blood vessels or axons through ECM interactions [120].

Research has revealed ECM-driven signals that shape tumor phenotypes and influence metabolic activity [121]. Moreover, the GB ECM is deficient in aggrecan, contains oncofetal proteins, and shows elevated levels of matrix metalloproteinases (MMPs), which increases ECM modulation, invasion, and angiogenesis [119,122]. The ECM’s significant role in cell survival, proliferation, and differentiation processes makes it an attractive target for novel therapeutic approaches, which could be particularly valuable given GB’s poor response to conventional treatments.

## 4. Nanotechnology in Glioblastoma

**Figure 3:**
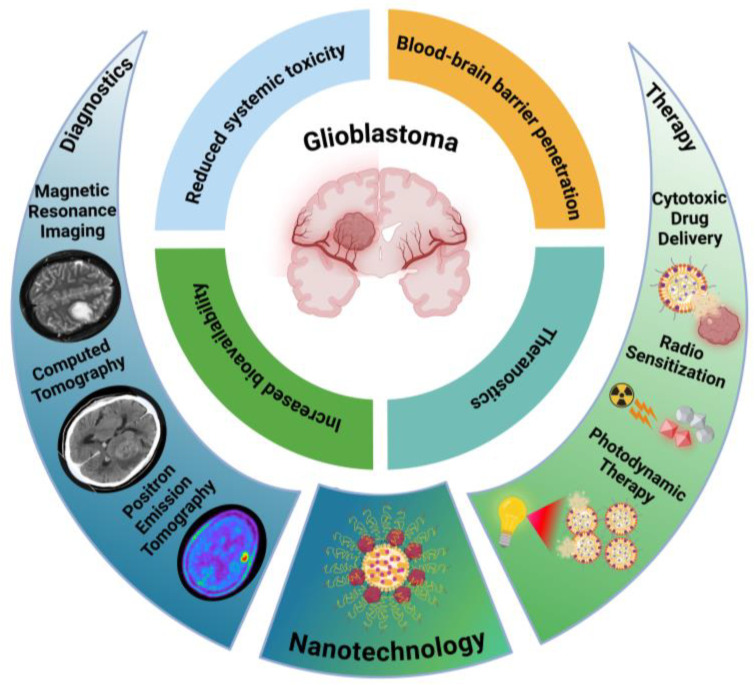
Nanotechnology benefits and examples of approaches in GB diagnostics and therapy. The brain images included in this figure were adapted from Gue, R. et al. [8], Mărginean, L. et al. [124], and Manzarbeitia-Arroba, B. et al. [125], and available under the Creative Commons Attribution (CC BY) license.

Among multiple alternative approaches to treat GB, nanotechnology has emerged as a particularly promising field to address the challenges posed by this tumor. Nanoparticle (NP)-based drug delivery systems offer the potential to overcome many of the challenges associated with treating brain tumors, including poor drug penetration across the BBB and the need for targeted delivery to tumor cells while minimizing toxicity to healthy tissue (Figure 3) [123].

Nanocarriers can be engineered to encapsulate various therapeutic agents, including chemotherapy drugs, small molecule inhibitors, nucleic acids for gene therapy, and even combinations of multiple agents for synergistic effects [126]. Their sizes typically range from 10 to 200 nm, with smaller sizes (10–50 nm) facilitating enhanced tumor penetration, cellular uptake, and TME diffusion, while larger sizes (100–200 nm) promote prolonged circulation, better retention at the tumor site, and reduced clearance through renal filtration [127].

Furthermore, the surface of nanoparticles can be modified with targeting ligands to facilitate transport across the BBB and to increase their specificity for GB cells [128]. As research in this area continues to evolve, nanotechnology-based approaches may offer new hope for improving the treatment outcomes for GB patients.

Despite all advances in this field, only a limited number of liposomal strategies have reached clinical trials in GB or other high grade gliomas, following a path similar to what was achieved in other cancers [129]. Among the main candidates studied, Caelyx™, a pegylated liposomal formulation of doxorubicin (DOX), underwent several phase I/II clinical trials, either alone or in a combination therapy (with TMZ, tamoxifen, RT, or a combination of these) [130,131,132,133,134]. However, these trials’ main conclusion was that the liposomal formulation did not show clinical benefits. More recently, a liposomal formulation of irinotecan (nal-IRI) was tested in two clinical trials, one as single agent and the other combined with TMZ; however, both trials ended due to lack of activity [135,136]. A new phase I clinical trial at Johns Hopkins University (NCT05768919) is currently recruiting patients to assess the efficacy of liposomal curcumin in combination with radiotherapy and TMZ; no study results have been released yet.

The limited efficacy observed thus far with these clinical trials suggests that while nanoparticle-based systems can improve drug delivery, further optimization in formulation and the development of new targeting strategies are necessary to enhance their clinical effectiveness in GB treatment. While nanotechnology offers promising advantages for GB treatment, its potential non-specific toxicity, both systemically and in brain tissue, remains a critical consideration. Ongoing research focuses on optimizing nanoparticle biocompatibility and biodegradability while minimizing inflammatory responses, aiming to optimize the balance between therapeutic efficacy and safety [137,138].

## 5. Nanotechnology and the ECM

**Figure 4:**
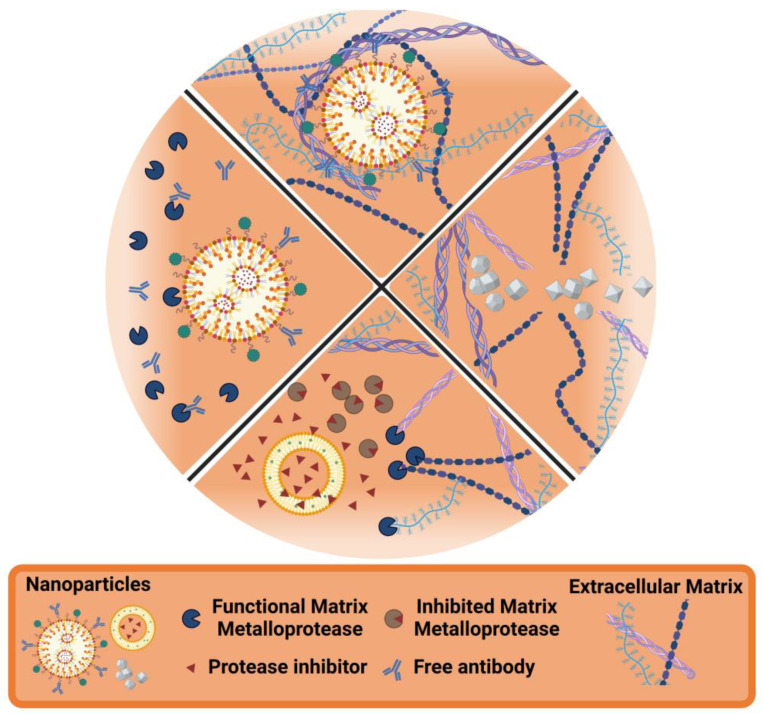
Nanotechnology strategies interacting with the ECM. Top: Nanoparticles targeting the ECM; Left: Nanoparticles responding to ECM cues (e.g., increased MMP concentration in the TME); Right: Nanoparticles degrading the ECM; Bottom: Nanoparticles preventing ECM degradation.

While initial nanotechnological approaches focused primarily on targeting glioma cells, there is growing recognition of the ECM as a critical factor to tumor progression and malignancy [114], as previously discussed. Consequently, researchers are developing novel therapeutic strategies that specifically target the ECM in GB. These exploit the ubiquitous presence of ECM components within the TME, and thus offer significant advantages compared to targeting the glioma cells themselves. These ECM-focused strategies, often investigated in preclinical settings, include the inhibition of matrix-degrading metalloproteinases and integrins, ECM stiffening, and degradation [139,140]. Additionally, targeting structural ECM molecules can enhance other treatment modalities, such as immunotherapy, by increasing the infiltration of immune cells [141,142]. Nanotechnology approaches interacting with the GB ECM can be categorized into the following classes, as exemplified in Figure 4: (i) ECM as the target: nanoparticles designed to directly interact with or modify the ECM structural components; (ii) ECM-responsive nanoparticles: nanoparticles designed to respond to specific ECM characteristics or changes; (iii) Enhancing ECM degradation: nanoparticles that facilitate the breakdown of ECM components to enhance drug penetration or disrupt tumor structure; (iv) Preventing ECM degradation: nanoparticles that inhibit matrix-degrading enzymes like metalloproteinases to maintain ECM integrity and limit tumor invasion.

Table 1 summarizes the various nanotechnology strategies discussed in this section, highlighting their approach, key features, and potential applications in GB treatment. This overview provides a comprehensive snapshot of the diverse nanotechnology approaches being explored to reprogram the GB ECM.

### 5.1. ECM as the Target

**Figure 5:**
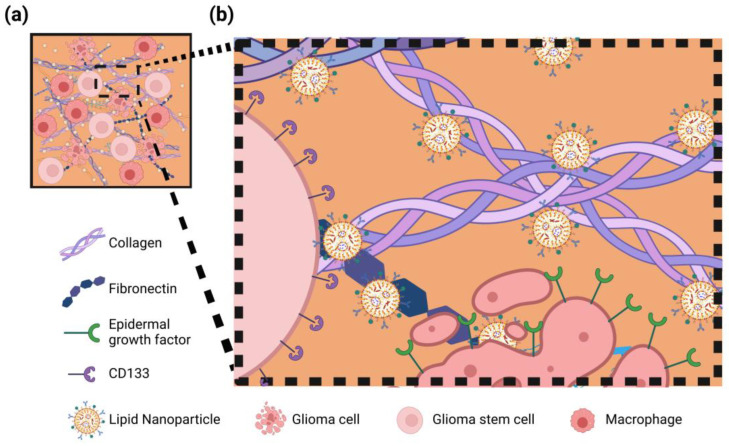
ECM targeting nanotechnology strategy. (**a**) Schematic overview illustrating nanoparticles interacting with ECM components to locally exert their effect; (**b**) Detailed depiction of nanoparticles avoiding cell receptors and specifically targeting ECM proteins such as collagen and fibronectin.

As previously mentioned, ECM components are ubiquitously distributed throughout the tumor parenchyma, making them a more consistent and accessible target compared to heterogeneous tumor cell populations (Figure 5) [141].

Among these components, the extra domain B (EDB) of fibronectin has emerged as a highly promising target for cancer therapy, particularly in aggressive solid tumors such as GB [162]. This specific domain is prominently present in the perivascular space of many aggressive tumors, making it an excellent marker for tumor-associated blood vessels [163]. Its unique overexpression pattern in the TME, coupled with its absence in normal adult tissues, makes EDB a selective target for nanotechnological strategies [164]. Saw et al. developed a GB therapy using a fibronectin-targeted liposomal nanoplatform functionalized with an EDB-specific aptide to deliver cyclophilin A (CypA), small interfering ribonucleic acid (siRNA). In vitro studies showed improved cellular uptake and CypA silencing in GB cells compared to non-targeted liposomes, while in non-orthotopic GB-xenografted mice, this targeted system reduced tumor growth and increased survival rates [143].

Previous studies have demonstrated that GB is characterized by a leaky and hemorrhagic vasculature, which promotes thrombosis and fibrin accumulation [144]. This feature, absent in normal tissues, is hypothesized to result from the increased permeability in GB, allowing plasma proteins to infiltrate the tumor tissue, where fibrinogen is transformed into fibrin through the action of pro-coagulant factors [165]. Therefore, fibrin deposition in the ECM, a feature of both primary and metastatic brain tumors, could be a promising target for therapeutic intervention.

Taking this into consideration, Chung et al. [144] developed peptide-functionalized CREKA-micelle nanoparticles to target fibrin deposits in GB [166]. In an orthotopic mouse model of GB, both targeted and non-targeted micelles accumulated at the tumor site due to the enhanced permeability and retention (EPR) effect. However, CREKA-micelles showed enhanced tumor homing within one hour, indicating active fibrin targeting. Biodistribution studies revealed micelle accumulation in the liver and kidneys, suggesting clearance through renal filtration and the reticuloendothelial system. Importantly, no cytotoxicity or tissue damage was observed, supporting the nanoparticle system’s safety [144].

Another promising ECM target in GB for tumor-specific therapy is TN-C, a glycoprotein with certain splice isoforms uniquely expressed in tumors. Clinical trials have evaluated radiolabeled antibodies targeting TN-C domains A1 and D for glioma and lymphoma therapy [167].

Instead of antibodies, Kang et al. developed a dual-targeting peptide system, Ft-PLA-PTX, which combines paclitaxel (PTX) with polylactic acid (PLA) nanoparticles. The peptide Ft targets TN-C and neuropilin-1 (NRP-1), enhancing internalization in U87 glioma and HUVEC cells, as well as penetration in 3D glioma spheroids. In vivo studies in U87 glioma-bearing mice showed significant accumulation of Ft-PLA-PTX at tumor sites, leading to higher cytotoxicity and apoptosis rates than single-targeted nanoparticles. Treatment resulted in a 269% increase in median survival compared to saline, outperforming Taxol^®^ and other formulations, indicating the effectiveness of this dual-targeting approach [145].

Lingasamy et al. studied the targeting of the same ECM molecules TN-C and NRP-1 using an 8-amino acid homing peptide, PL3, to enhance nanoparticle delivery to GB and prostate carcinoma. They found that PL3 effectively binds to both targets and functionalizing iron oxide nanoworms (NWs) and silver nanoparticles (AgNPs) with PL3 significantly improved their targeting ability in nude mice models, achieving an eight-fold increase in GB targeting compared to untargeted nanoparticles. Notably, PL3-guided NWs increased survival rates in glioma-bearing mice, while untargeted particles had no therapeutic effect. Furthermore, advanced imaging confirmed the accumulation of PL3-coated nanoparticles in TN-C and NRP-1 positive areas in clinical tumor samples, suggesting potential for future clinical applications [146]. Following their work with PL3, the authors investigated PL1, which targets the ECM components fibronectin EDB and TN-C. The PL1-functionalized AgNPs exhibited strong binding to these targets and facilitated cellular uptake in U87 cells via a macropinocytosis-like process. The binding affinity of PL1 was quantified, supporting its potential for delivering therapeutic agents intracellularly [147].

MMPs are key players in the ECM remodeling process that contributes to GB progression [168]. These zinc-dependent enzymes, essential for normal physiological processes, can contribute to malignancy when their regulation is disrupted [169]. In GB, several MMPs are overexpressed, with MMP-2 and MMP-9 being the most extensively studied [170,171,172]. These secreted gelatinases break down key ECM components like collagens, elastin, and fibronectin, creating space for tumor cell migration and invasion [173]. Additionally, they promote the release of pro-angiogenic factors such as VEGF, enhancing tumor resource availability and facilitating GB infiltration through newly formed blood vessels [174].

Another MMP found to be overexpressed in GB is the membrane-anchored MMP-14 (also known as MT1-MMP) [175]. It shares similar proteolytic, angiogenic, and immunomodulatory capabilities with the secreted gelatinases and can activate pro-MMP-2, further contributing to GB invasiveness [148,150]. MMP-14’s influence extends beyond ECM degradation, regulating numerous plasma membrane-anchored and extracellular proteins, thereby impacting both intercellular and cell-matrix communication [176]. In the GB TME, both cancer and stromal cells engage with the ECM through various adhesive structures [176]. This interaction often results in aggressive infiltration of adjacent brain tissue, including areas crucial for survival. To facilitate invasion, glioma cells secrete proteolytic enzymes that degrade the ECM and mediate the invasion process, and research has revealed that specific MMPs not only promote glioma cell invasion but also alter tumor cell behavior and stimulate cancer progression [118,177]. As the invasive nature of GB significantly contributes to its high mortality rate and poor prognosis, making it a critical area of study, understanding the complex interplay of various MMPs, in particular MMP-14, in GB is essential [175]. This deeper comprehension could lead to the development of new prognostic and predictive markers, improving patient outcomes. Moreover, exploring MMP interactions may pave the way for novel targeted therapies designed to counteract the invasive nature of GB.

Taking into consideration that MMP-14 is differentially expressed in GB, compared to normal tissues, Kasten et al. developed a dual-modality imaging agent targeting the membrane protease. Their peptide probe combines a near-infrared fluorescence (NIRF) dye linked to a quencher that is activated upon cleavage by MMP-14, and an MMP-14-binding peptide attached to a radionuclide chelate for positron emission tomography (PET). This approach enhanced tumor specificity and imaging contrast, as the NIRF signal activates only in the presence of MMP-14. In vitro and in vivo studies demonstrated the probe’s efficacy in detecting GB in cultured cell lines and patient-derived xenograft models, showing favorable tumor-to-background ratios. PET imaging confirmed significant localization of the probe in orthotopic GB models, and blocking experiments verified its specific targeting of MMP-14-expressing cells. Their strategy seemed to be effective for imaging GBs, providing valuable insights for both preoperative planning and real-time surgical guidance [148].

Finally, in terms of vascularization, GB stands out as one of the most highly angiogenic tumors, with VEGF playing a central role in this process [178]. The formation of new blood vessels in GB is driven by both hypoxia-dependent and independent mechanisms, leading to the development of unique vessels [109]. This angiogenic potential of GB is further enhanced by GSCs, which secrete significantly higher levels of VEGF compared to non-stem glioma cells [179]. Conditioned media from these stem cells have been shown to strongly enhance endothelial cell functions crucial for angiogenesis, including migration, proliferation, and tube formation. This heightened angiogenic activity, primarily driven by VEGF and exacerbated by GSCs, is a major contributor to the aggressive nature of GB, presenting both challenges and opportunities for therapeutic interventions targeting the tumor vasculature [180]. Taking advantage of the enhanced accumulation of VEGF in the ECM of GB tumors, Abakumov et al. developed iron oxide magnetic nanoparticles (MNPs) targeting VEGF for enhanced GB imaging [149]. The MNPs were coated with cross-linked bovine serum albumin (BSA) to improve biocompatibility and stability, then functionalized with monoclonal antibodies against VEGF, allowing specific binding to VEGF-positive GB cells. This targeted approach improved tumor visualization using magnetic resonance imaging (MRI), addressing limitations of existing contrast agents in specificity and retention [181]. In vitro studies confirmed the nanoparticles’ stability and cytocompatibility, while in vivo experiments in orthotopic C6-xenografted rat models demonstrated superior imaging of tumors and vasculature compared to non-targeted controls, with sustained contrast for 24 h post-injection [149].

### 5.2. ECM-Responsive Nanoparticles

**Figure 6:**
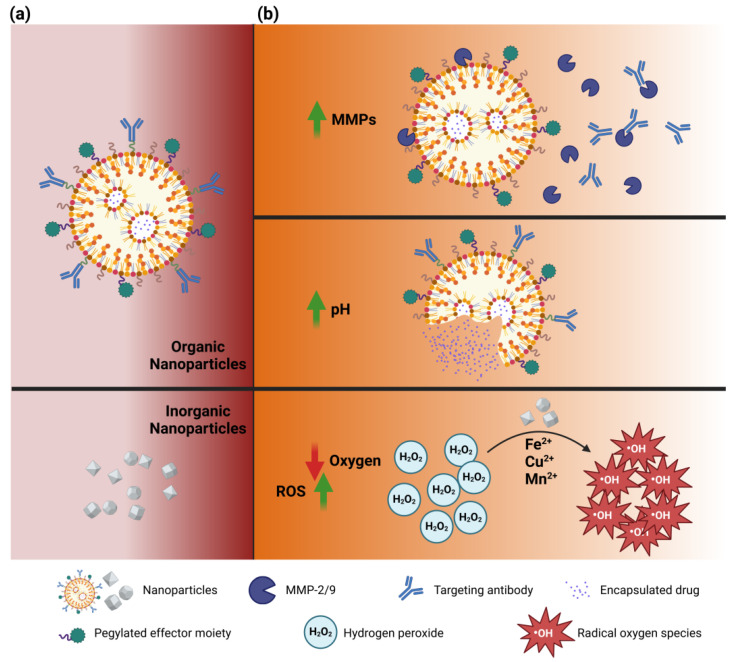
Utilizing ECM physicochemical cues as a nanotechnological strategy. (**a**) Nanoparticles in circulation; (**b**) Nanoparticles upon sensing TME conditional alterations. From top to bottom, an increase in secreted and membrane matrix metalloproteases, an increase in acidity, an increase in hypoxia accompanied by an increase in ROS.

The ECM provides essential mechanical and biochemical cues through its interactions with cell receptors, allowing it to function as both a structural scaffold and a regulator of cellular behaviors such as adhesion, proliferation, and differentiation [182]. The composition and architecture vary between tissue types, influenced by various factors including the cell type present, mechanical forces, and the availability of nutrients and oxygen in the local microenvironment. This dynamic nature of the ECM reflects changes in tissue status and disease conditions, with GB being no exception [183]. Nanosystems can be designed to modulate their effect once in contact with TME conditional changes, such as the increased presence of secreted and membrane MMPs, the acidic environment, and the lack of oxygenation (Figure 6).

As previously mentioned, MMP-14 is a proteolytic enzyme highly overexpressed in GBs and has been already applied as a microenvironment cue in GB therapy by Mohanty et al. Their work focused on creating a theranostic approach using cross-linked iron oxide nanoparticles, named CLIO-ICT, which combined therapeutic and diagnostic functions to target GB and GSCs. These nanoparticles consisted of cross-linked iron oxide particles conjugated to an azademethylcolchicine (ICT)-peptide prodrug that released the active drug upon cleavage by MMP-14. The study demonstrated that CLIO-ICT selectively targets GB cells and GSCs, disrupting tumor blood vessels and significantly inducing apoptosis while reducing GSCs populations. In vivo experiments in orthotopic xenografted mice revealed that CLIO-ICT, especially when combined with TMZ, improved survival rates and reduced tumor growth compared to TMZ alone. Additionally, the dual functionality of CLIO-ICT allowed for real-time tumor response monitoring via MRI. [150].

In a similar direction, Gu et al. [139] developed nanoparticles that utilize the upregulated expression of MMP-2 and MMP-9 in GB [168,184]. These nanoparticles were modified with an activatable cell-penetrating peptide, which is specifically cleaved by MMP-2 and MMP-9, enhancing internalization and enabling targeted PTX delivery within the GB microenvironment. After confirming successful conjugation of the peptide to polyethylene glycol-polycaprolactone (PEG-PCL) nanoparticles, in vitro studies showed significantly increased cellular uptake in GB cells and enhanced penetration in 3D GB spheroids. In vivo studies in intracranial GB xenograft models indicated that the peptide-modified nanoparticles accumulated in tumor tissues, leading to improved survival rates compared to conventional PTX formulations [139].

More recently, Fan et al. engineered MMP-2-activated nanoparticles that combine immunotherapy with ferroptosis induction for GB treatment [151]. These complex nanoparticles consist of bispecific antibodies targeting B7-H3 (associated with poor prognosis in GB) and CD3, along with a dimer of epigallocatechin-3-gallate (dEGCG) linked to HA via an MMP-2-cleavable sequence. dEGCG, on its own, has been shown to provide a myriad of antitumor effects, mainly associated with its production of intracellular ROS and subsequent induction of oxidative damage [185]. This design allowed the nanoparticles to cross the BBB, accumulate in GB tissue, and release their cargo upon MMP-2 cleavage. In vitro studies showed effective targeting of GB cells, T-cell activation, and cytokine release, leading to enhanced anti-tumor effects. In a mouse orthotopic U87 GB xenograft model, the nanoparticles demonstrated significantly higher intracranial accumulation compared to free antibodies or non-targeted formulations. Notably, the treatments resulted in 50% of mice surviving longer than 56 days, indicating a promising strategy for GB therapy that leverages both immune activation and ferroptosis [151].

In addition to MMP-responsiveness, tumor acidosis is a well-studied characteristic of most TMEs [186]. Rapidly proliferating tumor cells often deplete their own oxygen supply, thereby leading to hypoxia, where cells are unable to generate adenosine triphosphate (ATP) through oxidative phosphorylation. In response, the hypoxia-inducible factor (HIF) pathway shifts cellular metabolism towards anaerobic glycolysis, commonly referred to as the Warburg effect, allowing the tumor cells to continue ATP production despite the lack of oxygen. This metabolic adaptation results in the accumulation of lactate and protons as byproducts of fermentation, which alter not only the intracellular pH, but, once expelled, contribute to the characteristic acidification of the TME [94].

To take advantage of the acidic TME in GB, Zhao et al. developed a DOX-loaded liposome that responds to the surrounding pH by incorporating a conjugated peptide, which consists of a cell-penetrating sequence and a pH-sensitive trigger. The liposomes were produced through the common thin-film hydration method followed by the less common remote loading technique of DOX. In vitro experiments showed pH-triggered drug release and specific targeting of C6 and U87 GB cells under acidic conditions caused by the peptide modification. In vivo experiments in C6 subcutaneous and U87 orthotopic tumor-bearing mice demonstrated significant anti-tumor activity and antiangiogenic effects, indicating the liposomes’ potential for targeted drug delivery and vascular modulation [152].

In a simpler approach, Sathiyaseelan et al. utilized chitosan as the pH-responsive component in anti-nucleolin aptamer (AS1411)-functionalized gold nanoparticles, biosynthesized from *Gynura procumbens*, and loaded with both 5-fluorouracil (5-FU) and DOX. Chitosan, a cationic polysaccharide, becomes soluble at acidic pH, enhancing the release of its cargo in such environments. The study demonstrated higher drug release rates under acidic conditions that mimic the GB TME. In vitro experiments on LN229 GB cells showed that the dual-drug-loaded nanoparticles induced greater cell death than single-drug formulations, primarily through G0/G1 phase cell cycle arrest, with transmission electron microscopy (TEM) confirming their internalization in cell organelles [153].

More recently, our group has described an elegant functionalization design that utilizes an acid-cleavable angiopep-2 to functionally create two different nanosystems, a brain-targeting nanoparticle while in circulation that transforms into a GB-accumulating nanoparticle after BBB crossing [154]. In that sense, poly(lactic-*co*-glycolic acid) (PLGA) and PEG based NPs were designed to deliver docetaxel to GB tumors. The polymeric nanoparticles were dual-surface tailored with two key features: (i) a brain-targeted acid-responsive angiopep-2 moiety that triggers a structural rearrangement within BBB endosomal vesicles, and (ii) an L-histidine moiety that provides preferential accumulation into GB cells after BBB crossing. The angiopep-2 peptide targets the low-density lipoprotein receptor (LDLR) overexpressed on the BBB, while L-histidine targets the L-type amino acid transporter 1 (LAT1) overexpressed in GB cells. It is important to note that PEG with different molecular weights were used to create two levels of nanoparticle surface decoration, avoiding inter-steric hindrance. In vitro studies using tumor invasive margin patient cells showed that the stimuli-responsive multifunctional nanosystem effectively targeted GB cells, enhancing cell uptake 12-fold and inducing 3 times higher cytotoxicity in both 2D and 3D cell models compared to non-targeting formulations. In vitro BBB permeability was assessed with a hCMEC/D3 transwell model, with the nanosystem demonstrating a threefold increased BBB permeability. An in vivo biodistribution trial confirmed a threefold improvement of nanoparticle accumulation in the brain. The antitumor efficacy was validated in GB orthotopic models following both intratumoral and intravenous administration, with the median survival and number of long-term survivors increasing by 50% compared to control groups. The acid-cleavable properties of the nanoparticles ensure that the long-length PEG-angiopep-2 separates from the main structure after encountering the acidic pH of endosomes during BBB trafficking. This facilitates endosomal escape and nanoparticle expulsion towards the brain. Subsequently, the shorter PEG-L-histidine becomes exposed on the NP surface due to steric deprotection, allowing for GB cell targeting. This study represented the first time that a multi-ligand functionalized NP system was proposed to sequentially target the BBB and GB through the exploitation of a BBB-responsive transport mechanism [154].

Furthermore, as previously noted, hypoxia is a prevalent feature in cancer. The TME often stimulates neoangiogenesis, resulting in the development of aberrant and inefficient blood vessels. This process, paradoxically, intensifies the hypoxic conditions within the tumor. These irregular vessels restrict the delivery of oxygen, drugs, and immune cells, making tumors more resistant to treatment. Hypoxia also induces significant changes in tumor cells, activating pathways such as the previously mentioned HIF pathway, which enables cell adaptation, promoting invasion, therapy resistance, and immune evasion [187]. This is particularly relevant in GB, one of the most hypoxic tumors [188]. Very recently, Qi et al. developed a hypoxia-responsive liposomal system (AMVY@NPs) for GB treatment, designed for stepwise targeting and drug release. This system includes a polymetronidazole core that encapsulates the Yes-associated protein (YAP) inhibitor verteporfin (VP), a cationic lipid layer that adsorbs siRNA targeting YAP (siYAP), and an outer coating with the targeting peptide angiopep-2. The nanoparticles are engineered to cross the BBB, specifically target GB cells, and release their cargo in response to the hypoxic TME. In vitro and in vivo studies demonstrated that AMVY@NPs effectively released siYAP and VP under hypoxic conditions, leading to synergistic inhibition of GB cell growth and pluripotency. In an orthotopic U87 xenograft mouse model, these nanoparticles significantly inhibited tumor growth and improved survival without noticeable adverse effects [155].

TME hypoxia and acidosis are linked to ROS creation by oxidative stress [189]. In GB, elevated ROS levels are a hallmark of the TME, promoting tumor progression, therapy resistance, genomic instability, and invasion [190]. While therapies such as radiation aim to exploit ROS-induced cell death, GB cells often resist by enhancing antioxidant defenses [191]. Considering this, Yang et al. developed a hollow manganese dioxide (h-MnO_2_) nanosystem aimed at alleviating tumor hypoxia by degrading endogenous hydrogen peroxide (H_2_O_2_) into oxygen and water. The pH-responsive MnO_2_ breaks down in the acidic TME. This nanosystem, produced via a silica template method and functionalized with PEG for stability, enables targeted drug delivery, imaging, and TME modulation. It efficiently loaded chlorine e6 (Ce6), a photodynamic agent, and DOX, releasing them in the TME while generating Mn^2+^ ions to enhance MRI contrast. By producing oxygen, the system improves photodynamic therapy efficacy, which relies on oxygen to generate ROS. In vivo studies confirmed effective accumulation of Ce6/DOX-loaded nanoparticles in tumors and kidneys, demonstrating synergistic effects of chemotherapy and photodynamic therapy in reducing tumor mass [156].

Finally, regarding ECM-responsiveness, the interaction between glutathione (GSH) and disulfide (SS) bonds has been exploited [192]. This interaction relies on two characteristics: (i) there is a steep GSH gradient between the extracellular environment (micromolar) and the intracellular tumor environment (millimolar), and (ii) SS bonds can be cleaved by GSH. Therefore, drug delivery systems that incorporate SS bonds can rapidly release their therapeutic agents inside target cells, significantly improving drug efficacy [157,193].

This was explored by Tian et al., who developed a redox-sensitive nanocarrier system for targeted curcumin delivery to GB cells by conjugating HA with curcumin via disulfide bonds. This system takes advantage of the high glutathione levels in TMEs, where GSH can cleave disulfide bonds, leading to rapid drug release inside target cells. Their work explored how the molecular weight (MW) of HA affects the properties and performance of nanocarriers. The HA-curcumin conjugates formed nanoscale micelles, enhancing curcumin’s solubility and stability. Low (50 kDa) and medium (200–500 kDa) MW HA-based micelles showed GSH sensitivity and superior cytotoxicity and cellular uptake in G422 mouse GB cells compared to high MW micelles or free curcumin, indicating their effectiveness for intracellular drug delivery [157].

### 5.3. Enhancing ECM Degradation

The GB ECM, as previously discussed, is notably dense and complex, being comprised of overexpressed components, including HA, specific collagens, laminin, TN-C, and fibronectin, among others (Figure 2). These excessive ECM components make the TME more compact and less flexible, hindering the diffusion of therapeutic agents [194]. Additionally, this dense physical barrier impedes both T cell infiltration and drug or nanosystem penetration, contributing to immunosuppression and drug resistance [195]. Recent studies suggest that breaking down the tumor ECM or inhibiting its formation can enhance the penetration and accumulation of nanotherapeutics, thereby improving the efficacy of nanomedicines [140]. One approach to address this issue involves degrading key ECM components, which loosens the ECM structure and increases tumor tissue permeability, allowing immune cells to better migrate and infiltrate the tumor [196].

To optimize the ECM degradation strategy by targeting the most abundant components of GB ECM, Kiyokawa et al. investigated the role of HA degradation in enhancing the effect of oncolytic adenovirus immunotherapy for GB [158]. They used ICOVIR17, a hyaluronidase-expressing oncolytic adenovirus, to target the HA-rich ECM. In murine orthotopic GB models, ICOVIR17 increased tumor-infiltrating CD8^+^ T cells and macrophages, leading to prolonged animal survival compared to the control virus ICOVIR15 (no hyaluronidase). ICOVIR17 also upregulated programmed cell death protein (PD)-ligand 1 (PD-L1) expression on GB cells and macrophages. In vitro experiments showed that high MW HA inhibited adenovirus-induced NF-kB signaling in macrophages, linking HA degradation to macrophage activation. Combining ICOVIR17 with anti-PD-1 antibody therapy further extended survival in GB mice models, with some animals achieving long-term remission. Mechanistic studies revealed that CD4^+^ T cells, CD8^+^ T cells, and macrophages all contributed to the efficacy of the combination therapy. This treatment induced pro-inflammatory TAMs and enhanced tumor-specific T cell cytotoxicity both locally and systemically [158].

Similarly, Shukla et al. addressed ECM as barrier to nanoparticle penetration through the functionalization of human serum albumin nanoparticles (HSANPs) with collagenase. These collagenase-modified nanoparticles were loaded with gemcitabine (CG-HSANPs) to overcome the limitations of its short half-life and dose-dependent side effects. In vitro, CG-HSANPs demonstrated comparable cytotoxicity with uncoated NPs or free drug but exhibited improved tumor spheroid penetration. Also, CG-HSANPs induced higher levels of nuclear fragmentation, ROS generation, and mitochondrial membrane potential disruption compared to the native drug, emphasizing the importance of ECM degradation in enhancing drug delivery efficacy [159].

### 5.4. Preventing ECM Degradation

The invasive nature of GB is closely associated with its ECM, which facilitates tumor cell migration and invasion [176]. As previously elucidated, given the role of MMPs in GB progression, various approaches targeting their inhibition have been explored [197,198]. Synthetic MMP inhibitors such as Batimastat and Marimastat, which bind to the MMPs’ zinc atom, have shown promise in reducing MMP activity [199]. However, clinical studies have failed to demonstrate significant improvements in patient progression-free and overall survival, primarily due to the molecules’ low aqueous solubility and potential side effects [200]. As a result, research focusing on MMPs and ECM remodeling as actively targeted therapeutic strategies has been relatively limited [201]. Despite these past challenges, recent strategies have sought innovative ways to target MMPs. While traditional hydroxamate-based MMP inhibitors faced clinical setbacks, nanotechnology offers a promising alternative. By improving solubility and minimizing side effects, fields where nanoparticles excel, nanosystems have the potential to reinvigorate these inhibitors and enable more effective ECM-targeted therapies.

The scorpion venom-derived chlorotoxin (CTX) is a more modern and less synthetic alternative for the old hydroxamate-based MMP inhibitors [202] and has demonstrated active targeting of GB cells [203]. Agarwal et al. functionalized PLGA nanoparticles with CTX (with high affinity for MMP-2 and chloride channels overexpressed in GB cells) to deliver morusin, a chemotherapeutic agent with poor bioavailability. These CTX-functionalized nanoparticles (PLGA-MOR-CTX) effectively crossed the BBB and presented anti-cancer effects in U87 and GI-1 human GB cell lines through apoptosis induction, ROS generation, and MMP inhibition, with low toxicity to normal neuronal cells [160].

Islam et al. synthesized amphiphilic peptides containing a brain-targeting ligand (HAIYPRH or CKAPETALC) conjugated with an MMP-9 inhibiting peptide (CTTHWGFTLC), linked by glycine spacers and conjugated to cholesterol at the N-terminus [161]. The amphiphilic peptide-cholesterol (c-GGGCTTHWGFTLCHAIYPRH) formed nanoparticles able to cross an in vitro BBB hCMEC/D3 transwell model while showing low toxicity in HeLa cells. The authors also demonstrated an efficient inhibition of MMP-9 activity in an acellular in vitro setting [161].

## 6. Challenges and Future Perspectives

Nanotechnology offers significant promise to address the challenges of GB therapy, particularly in targeting its ECM. However, several hurdles remain to be addressed to achieve its full clinical potential.

The GB ECM is highly complex and heterogeneous, varying between patients and even within the same tumor. This makes it difficult to develop a one-size-fits-all nanoparticle approach. Additionally, the dense and rigid structure of the GB ECM poses a significant barrier to nanoparticle penetration and drug delivery. Current ECM degradation strategies need further enhancement. Additionally, the BBB presents a major obstacle. While some promising nanoparticle targeting strategies have shown promise in preclinical models, translating these results to humans remains challenging due to differences in BBB structure and function.

Current research is exploring innovative alternatives to address these issues. Multi-functional nanoparticles are being designed to simultaneously target GB cells and modulate ECM. For example, particles incorporating MMP inhibitors alongside chemotherapeutic agents aim to remodel the ECM and expose tumor cells to treatment. Stimuli-responsive systems, which activate in response to specific TME cues (e.g., pH, enzyme activity), are another promising avenue. This approach could improve target specificity by guaranteeing that the effect only occurs within the TME and reduce off-target effects. Biomimetic strategies, such as nanoparticles coated with cell membranes or engineered to mimic extracellular vesicles, offer potential to improve BBB crossing and tumor targeting. Combination therapies, like using nanoparticles in conjunction with other treatment modalities, such as focused ultrasound to temporarily disrupt the BBB, could enhance delivery to the tumor site.

Looking forward, several key areas of research are essential for advancing this field. Personalized approaches, such as tailoring NPs based on the ECM of each individual patient, could greatly improve therapeutic outcome by tailoring nanoparticle formulations accordingly. Advanced imaging techniques could enable real-time visualization of nanoparticle distribution and ECM interactions in vivo, optimizing delivery strategies. Robust predictive in vitro and in silico models of the GB ECM and BBB are needed to accelerate nanoparticle design and testing. High-throughput screening of NP formulations generated by these predictive models could identify the most feasible designs. Additionally, long-term safety studies are crucial to evaluate the potential long-term impact of ECM-modulating nanoparticles on healthy brain tissue and tumor recurrence. Ultimately, scalable manufacturing, by developing cost-effective and reproducible methods for large-scale production of complex nanoparticle formulations, will invariably have to be implemented.

In conclusion, nanotechnology offers promising avenues for advancing GB treatment by addressing key challenges such as BBB penetration, targeted drug delivery, and overcoming drug resistance. The ability to modulate the ECM through nanotechnological approaches presents a particularly exciting avenue for therapeutic strategies. While significant hurdles remain, including scale-up optimization and reproducibility, the rapid progress in this field provides cautious optimism for developing more effective GB therapies in the coming decade. As we continue to unravel the complexities of GB biology and refine nanotechnological tools, the potential for significant improvements in patient outcomes becomes ever more tangible.

## Figures and Tables

**Table 1:** Overview of nanotechnology approaches for modulating the ECM in GB.

Strategy	Approach	Key Features	Outcome	Ref.
ECM as the target	Fibronectin-targeted liposomal nanoplatform	Functionalized with EDB-specific aptide to deliver CypA siRNA	Improved cellular uptake and CypA silencing in GB cells; reduced tumor growth and increased survival rates in vivo	[143]
	CREKA-micelle nanoparticles	Targets fibrin deposits in GB	Enhanced tumor homing; potential for targeted drug delivery	[144]
	Ft-PLA-PTX dual-targeting peptide system	Targets TN-C and NRP-1	Enhanced internalization in glioma cells and penetration in 3D spheroids; Increased median survival compared to saline	[145]
	PL3-functionalized iron oxide nanoworms and silver nanoparticles	Targets TN-C and NRP-1	Increased GB targeting; increased survival rates in glioma-bearing mice	[146]
	PL1-functionalized silver nanoparticles	Targets fibronectin EDB and TN-C	Strong binding to targets and facilitated cellular uptake	[147]
	MMP-14 targeting dual-modality imaging agent	Combines NIRF dye and PET radionuclide	Enhanced tumor specificity and imaging contrast for GB detection; potential for preoperative planning and real-time surgical guidance	[148]
	VEGF-targeting iron oxide magnetic nanoparticles	Coated with cross-linked BSA and functionalized with anti-VEGF antibodies	Improved tumor visualization using MRI; sustained contrast for 24 h post-injection	[149]
ECM-responsive nanoparticles	CLIO-ICT nanoparticles	MMP-14-activated prodrug release system	Selective targeting of GB cells and GSCs; disrupted tumor blood vessels; induced apoptosis and reduced GSCs populations; real-time tumor response monitoring via MRI	[150]
	Activatable cell-penetrating peptide-modified nanoparticles	MMP-2 and MMP-9 responsive design	Enhanced internalization and targeted PTX delivery within GB microenvironment; improved penetration in 3D GB spheroids; increased survival rates compared to conventional PTX formulations	[139]
	MMP-2-activated nanoparticles with bispecific antibodies	Combines immunotherapy with ferroptosis induction	Targeted B7-H3 and CD3; crossed BBB; accumulated in GB tissue; released cargo upon MMP-2 cleavage; activated T-cells and induced cytokine release	[151]
	pH-responsive DOX-loaded liposomes	Incorporates pH-sensitive peptide trigger	pH-triggered drug release; specific targeting of GB cells under acidic conditions; anti-tumor activity and anti-angiogenic effects	[152]
	Chitosan-based pH-responsive gold nanoparticles	Functionalized with anti-nucleolin aptamer (AS1411)	Enhanced drug release in acidic environments; dual-drug delivery (5-FU and DOX); induced greater cell death than single-drug formulations	[153]
	Acid-cleavable angiopep-2 functionalized nanoparticles	Dual-surface tailored PLGA and PEG based NPs	Brain-targeting in circulation; transformed into GB-accumulating after BBB crossing; enhanced cell uptake and cytotoxicity; improved BBB permeability	[154]
	Hypoxia-responsive liposomal system (AMVY@NPs)	Stepwise targeting and drug release	Crossed BBB; targeted GB cells; released cargo in hypoxic conditions; synergistic inhibition of GB cell growth and pluripotency	[155]
	Hollow manganese dioxide (h-MnO_2_) nanosystem	pH-responsive design	Alleviated tumor hypoxia; enabled targeted drug delivery, imaging, and TME modulation; enhanced photodynamic therapy efficacy	[156]
	Redox-sensitive HA-curcumin conjugate nanocarriers	GSH-responsive disulfide bonds	Rapid drug release in high GSH environments; enhanced curcumin solubility and stability; superior cytotoxicity and cellular uptake in GB cells	[157]
Enhancing ECM degradation	Hyaluronidase-expressing oncolytic adenovirus (ICOVIR17)	Targets HA-rich ECM; increases tumor-infiltrating CD8^+^ T cells and macrophages	Prolonged survival in GB models; potential combination with anti-PD-1 therapies for enhanced efficacy	[158]
	Collagenase-modified human serum albumin nanoparticles (CG-HSANPs)	Loaded with gemcitabine; improved tumor spheroid penetration	Overcame limitations of gemcitabine’s short half-life; enhanced drug delivery efficacy and induced higher levels of nuclear fragmentation and ROS generation	[159]
Preventing ECM degradation	PLGA nanoparticles functionalized with chlorotoxin (CTX)	Targets MMP-2 and chloride channels overexpressed in GB cells; delivers morusin	Effectively crossed BBB; induced apoptosis and ROS generation in GB cells with low toxicity to normal cells	[160]
	Amphiphilic peptide nanoparticles	Conjugated with MMP-9 inhibiting peptide; brain-targeting ligand included	Efficiently inhibited MMP-9 activity; demonstrated low toxicity while crossing the BBB	[161]

## Data Availability

No new data were created or analyzed in this study. Data sharing is not applicable to this article.

## Abbreviations

The following abbreviations are used in this manuscript:

GB	Glioblastoma
CNS	Central Nervous System
WHO	World Health Organization
IDH	Isocitrate Dehydrogenase
TERT	Telomerase Reverse Transcriptase
EGF	Epithelial Growth Factor
EGFR	Epithelial Growth Factor Receptor
NOS	Not Otherwise Specified
NEC	Not Elsewhere Classified
RT	Radiotherapy
TMZ	Temozolomide
MGMT	Methylguanine Methyltransferase
FDA	Food and Drug Administration
VEGF	Vascular Endothelial Growth Factor
BBB	Blood-Brain Barrier
TTFields	Tumor Treating Fields
TME	Tumor Microenvironment
TCGA	The Cancer Genome Atlas
CDKN2A	Cyclin-Dependent Kinase Inhibitor 2A
TP53	Tumor Protein P53
SMO	Smoothened
GAS1	Growth Arrest-Specific Protein 1
GLI2	GLI family zinc finger 2
NOTCH3	Neurogenic Locus Notch Homolog Protein 3
JAG1	Jagged 1
LFNG	Beta-1,3-*N*-acetylglucosaminyltransferase Lunatic Fringe
NF1	Neurofibromin
PTEN	Phosphatase and Tensin Homolog
PI3K	phosphatidylinositol 3-kinase
CHI3L1	Chitinase-3-Like Protein 1
CD	Cluster of Differentiation
NF-κB	Nuclear Factor Kappa-Light-Chain-Enhancer of Activated B Cells
PDGFRA	Platelet-Derived Growth Factor Receptor A
AC-like	Astrocyte-like
MES-like	Mesenchymal-like
OPC-like	Oligodendrocyte Progenitor-like
NPC-like	Neural Progenitor-like
CDK4	Cyclin-Dependent Kinase 4
GSCs	Glioma Stem Cells
SOX2	Sex Determining Region Y Box 2
DNA	Deoxyribonucleic Acid
ABC	ATP-Binding Cassette
OCT4	Octamer-Binding Transcription Factor 4
IL	Interleukin
TGF-β	Transforming Growth Factor Beta
TAM	Tumor-Associated Macrophage
CSF-1	Colony Stimulating Factor 1
GDNF	Glial Cell Line-Derived Neurotrophic Factor
CCL2	Chemokine (CC Motif) Ligand 2
SFD-1	Stromal Cell-Derived Factor 1
CXCL1	Chemokine (CXC Motif) Ligand 1
GM-CSF	Granulocyte-Macrophage Colony-Stimulating Factor
IGF-1	Insulin-like Growth Factor 1
PDGF	Platelet-Derived Growth Factor
MDSC	Myeloid-Derived Suppressor Cells
NK	Natural Killer
ROS	Reactive Oxygen Species
CAF	Cancer-Associated Fibroblast
ERK	Extracellular Signal-Regulated Kinase
ECM	Extracellular Matrix
HA	Hyaluronic Acid
TN-C	Tenascin-C
MMP	Matrix Metalloproteinase
NP	Nanoparticle
DOX	Doxorubicin
EDB	Extra Domain B
CypA	Cyclophilin A
siRNA	Small Interfering Ribonucleic Acid
EPR	Enhanced Permeability and Retention
PTX	Paclitaxel
PLA	Polylactic Acid
NRP-1	Neuropilin-1
NW	Nanoworm
AgNP	Silver Nanoparticle
NIRF	Near-Infrared Fluorescence
PET	Positron Emission Tomography
MNP	Magnetic Nanoparticle
BSA	Bovine Serum Albumin
MRI	Magnetic Resonance Imaging
CLIO	Cross-Linked Iron Oxide
ICT	Azademethylcolchicine
PEG	Polyethylene Glycol
PCL	Polycaprolactone
dEGCG	Epigallocatechin-3-Gallate Dimer
ATP	Adenosine Triphosphate
HIF	Hypoxia-Inducible Factor
AS1411	Anti-Nucleolin Aptamer
5-FU	5-Fluorouracil
TEM	Transmission Electron Microscopy
PLGA	Poly(Lactic-Co-Glycolic Acid)
LDLR	Low-Density Lipoprotein Receptor
LAT1	L-type Amino Acid Transporter 1
YAP	Yes-Associated Protein
VP	Verteporfin
h-MnO_2_	Hollow Manganese Dioxide
H_2_O_2_	Hydrogen Peroxide
Ce6	Chlorine e6
GSH	Glutathione
SS	Disulfide Bonds
MW	Molecular Weight
PD	Programmed Cell Death Protein
PD-L1	Programmed Death-Ligand 1
CG-HSANP	Collagenase-Modified Human Serum Albumin Nanoparticle
CTX	Chlorotoxin
